# Programming of Dopaminergic Neurons by Neonatal Sex Hormone Exposure: Effects on Dopamine Content and Tyrosine Hydroxylase Expression in Adult Male Rats

**DOI:** 10.1155/2016/4569785

**Published:** 2016-01-10

**Authors:** Pedro Espinosa, Roxana A. Silva, Nicole K. Sanguinetti, Francisca C. Venegas, Raul Riquelme, Luis F. González, Gonzalo Cruz, Georgina M. Renard, Pablo R. Moya, Ramón Sotomayor-Zárate

**Affiliations:** ^1^Laboratorio de Neuroquímica y Neurofarmacología, Centro de Neurobiología y Plasticidad Cerebral, Instituto de Fisiología, Facultad de Ciencias, Universidad de Valparaíso, 2360102 Valparaíso, Chile; ^2^Laboratorio de Neurogenética, Centro de Neurobiología y Plasticidad Cerebral, Instituto de Fisiología, Facultad de Ciencias, Universidad de Valparaíso, 2360102 Valparaíso, Chile; ^3^Laboratorio de Alteraciones Reproductivas y Metabólicas, Centro de Neurobiología y Plasticidad Cerebral, Instituto de Fisiología, Facultad de Ciencias, Universidad de Valparaíso, 2360102 Valparaíso, Chile

## Abstract

We sought to determine the long-term changes produced by neonatal sex hormone administration on the functioning of midbrain dopaminergic neurons in adult male rats. Sprague-Dawley rats were injected subcutaneously at postnatal day 1 and were assigned to the following experimental groups: TP (testosterone propionate of 1.0 mg/50 *μ*L); DHT (dihydrotestosterone of 1.0 mg/50 *μ*L); EV (estradiol valerate of 0.1 mg/50 *μ*L); and control (sesame oil of 50 *μ*L). At postnatal day 60, neurochemical studies were performed to determine dopamine content in substantia nigra-ventral tegmental area and dopamine release in nucleus accumbens. Molecular (mRNA expression of tyrosine hydroxylase) and cellular (tyrosine hydroxylase immunoreactivity) studies were also performed. We found increased dopamine content in substantia nigra-ventral tegmental area of TP and EV rats, in addition to increased dopamine release in nucleus accumbens. However, neonatal exposure to DHT, a nonaromatizable androgen, did not affect midbrain dopaminergic neurons. Correspondingly, compared to control rats, levels of tyrosine hydroxylase mRNA and protein were significantly increased in TP and EV rats but not in DHT rats, as determined by qPCR and immunohistochemistry, respectively. Our results suggest an estrogenic mechanism involving increased tyrosine hydroxylase expression, either by direct estrogenic action or by aromatization of testosterone to estradiol in substantia nigra-ventral tegmental area.

## 1. Introduction 

Different adverse stimuli in early life produce alterations in normal development that persist until adulthood and may be risk factors for diseases in adult life. Lucas defined the concept of* programming* as “the physiological redirection of a tissue or organ by a stimulus, in a sensitive period of development, that produces adverse functional changes in adulthood” [1]. Animal research initially focused on fetal exposure (*fetal programming*), but recent research has expanded the concept of programming to include early postnatal exposure (*neonatal programming*).

In 1959, Phoenix et al. [2] first reported the long-term effects produced by androgens on the central nervous system (CNS) and their behavioral implications in reproduction [2]. This early research determined that the maximum sensitivity to the effects of androgens occurs during the gestational period, childhood, and puberty [3, 4]. In recent years, it has been shown that environmental pollutants act as endocrine disruptors capable of producing a myriad of effects in the brain (for review see [5]). For example, some pollutants, such as polychlorinated biphenyls (administered in neonatal period, through lactation), produce learning deficits and changes in spatial orientation tasks in monkeys and rats (for review see [6]). Moreover, neonatal exposure to polybrominated diphenyl ether (PBDE), another endocrine disrupter, increases spontaneous locomotor behavior in adult rats [7]. Interestingly, the authors showed that different doses of PBDE administered during neonatal period can result in either an increase or decrease in nicotine-induced locomotor activity [7]. Exposure to environmental pollutants affects specific neuronal groups including midbrain dopaminergic neurons. For example, neonatal or postnatal administration of bisphenol A (BPA) in rats produces an increase in spontaneous locomotion behavior, which was associated with decreased immunoreactivity for tyrosine hydroxylase (TH) in substantia nigra (SN) and a decrease in gene expression of dopamine transporter (DAT) in midbrain nuclei [8]. Therefore, the evidence in the literature shows that the neonatal period is a window of sensitivity to the effects of hormonally active compounds, when exposure to compounds such as endocrine disrupter or sex hormones can generate long-term effects on the functioning of neural circuits.

In CNS, the physiological effects of testosterone (T) are mediated through its reduction to dihydrotestosterone (DHT) by cytochrome P450 5-*α*-reductase [9] or its aromatization to estradiol (E_2_) by cytochrome P450 aromatase [10]. In the brain, cytochrome P450 5-*α*-reductase exists in two isoforms [11, 12]; type-1 isoform is expressed in similar levels in females and males [12], while the type-2 isoform is only expressed in males in the late stage of fetal development and early stage of postnatal period [10]. On the other hand, cytochrome P450 aromatase is highly expressed in males in the hypothalamus during gestational development and then progressively decreases during the neonatal stage and through childhood and adulthood [10]. The same pattern is observed for cytochrome P450 aromatase activity in hypothalamus of male rats [13]; however, in the female brain, cytochrome P450 aromatase activity is steady throughout all life stages [13]. Interestingly, in midbrain, cytochrome P450 aromatase activity is highest during the first two weeks of neonatal stage in both male and female rats [13].

Dopaminergic neurons are one of the major groups of cells in the midbrain and belong to nigrostriatal and mesocorticolimbic circuits [14]. The nigrostriatal pathway is formed by dopaminergic projections from the SN* pars compacta* to the striatum [15–18], while dopaminergic projections from ventral tegmental area (VTA) to nucleus accumbens (NAcc) and prefrontal cortex (PFC) form the mesocorticolimbic circuit, or reward system [19–22]. In the latter, natural rewarding cues such as sex [23] or food [24], as well as drugs of abuse, [25] increase extracellular dopamine (DA) levels in NAcc.

Sex hormone receptors are expressed in both nigrostriatal and mesocorticolimbic circuits [26–30] and regulate the expression of critical proteins for dopaminergic function, such as tyrosine hydroxylase (TH), the rate-limiting enzyme of catecholamine synthesis [29, 31–34]. Sex hormone exposure during early developmental stages or sensitive period of life has been associated with some CNS disorders. In humans, research has shown that increased androgen levels during adolescence are associated with risky behavior, such as development and maintenance of alcohol dependence [35]. Also, studies have shown that increased levels of sex hormone in amniotic fluid during the fetal stage are positively related with autism [36]. Animal studies have shown that androgen exposure during early development might play a role in the development of attention deficit hyperactivity disorder [37]. At neurochemical level, neonatal testosterone exposure decreases extracellular serotonin levels in the amygdala [38] and increases noradrenaline (NA) and glutamate in ventromedial hypothalamus [39] of adult female rats. In regard to estrogens, we previously found that neonatal exposure to estradiol valerate (EV) in female rats increases DA content in the tuberoinfundibular area [39] and nigrostriatal pathway [40] in adulthood. However, the long-lasting effects of neonatal exposure to different sex hormones on midbrain dopaminergic neurons have been scarcely studied. Thus, the aim of the present study was to determine potential changes induced by neonatal administration of testosterone propionate (TP), DHT, or EV on DA content and expression of TH mRNA in midbrain dopaminergic neurons in adult male rats.

## 2. Materials and Methods

### 2.1. Animals

93 male Sprague Dawley pups from fifteen litters were used. The remaining female pups were assigned to other studies. All animals were housed in a temperature-controlled room (21 ± 2°C) under a 12 h light cycle with lights on at 08:00 h, with food and water* ad libitum*. All experimental procedures were approved by Ethics Committee of the Faculty of Science, Universidad de Valparaíso, and the Institutional Animal Experimentation Ethics Board and the Science Council (FONDECYT) of Chile. Efforts were made to minimize the number of animals used and their suffering.

### 2.2. Drugs and Reagents

Testosterone propionate (TP), dihydrotestosterone (DHT), estradiol valerate (EV), sesame oil, dopamine standard, EDTA, and 1-octanesulfonic acid were purchased from Sigma-Aldrich, Inc. (St. Louis, Missouri, USA). All other reagents were of analytical and molecular grade.

### 2.3. Experimental Procedure

Each group of animals was single-injected at postnatal day (PND) 1 with 1.0 mg TP, 1.0 mg DHT, 0.1 mg EV, or sesame oil (control) per pup. TP, DHT, and EV were dissolved in 50 *μ*L of sesame oil. Pups were divided randomly into four groups of animals: control (*n* = 29), TP (*n* = 32), DHT (*n* = 27), and EV (*n* = 32). The doses of TP, DHT, and EV used were previously published [39, 40, 42, 43]. All the pups were raised with a lactating mother until the weaning age at PND 21. After weaning, animals were housed in groups according to gender and treatment in standard cages. At PND 60, the groups of rats were randomly assigned for the following experimental protocols.

*(i) Determination of DA Content*. Rats were decapitated with a guillotine and the brain was removed. We microdissected at 4°C SN and VTA (which were dissected as one tissue) and striatum; these brain tissues were weighed on analytical balance (model JK-180, Chyo, Japan) as previously described [40, 44, 45]. Brain tissues were stored at −80°C for further analysis. 

*(ii) Determination of TH mRNA*. Rats were decapitated and the brain was removed. SN and VTA were separately microdissected at 4°C using micropunch (Harris Micro-Punch, tip diameter of 2.0 mm, Ted Pella Inc., CA, USA). Brain tissues were weighed on an analytical balance and stored at −80°C for further analysis. 

*(iii) Determination of TH Protein*. Rats were anesthetized and transcardially perfused. Brains were removed and cut into coronal slices for immunohistochemistry for TH. 

*(iv) Determination of NAcc DA Release*. Using* in vivo* brain microdialysis in anesthetized animals, basal and stimulated-K^+^ DA extracellular levels were measured through HPLC coupled to electrochemical detection. After completion of* in vivo* experiments, rats were euthanized by decapitation.

### 2.4. Real-Time PCR

Real-time PCR was used to determine whether the mRNA encoding TH changed in the SN and VTA in adulthood of male rats exposed to sex hormones at PND 1. Total RNA was extracted using RNeasy Mini Kit (number 74104, Qiagen, Valencia, CA, USA) following manufacturer instructions. The quantification of total RNA was made in NanoDrop ND-1000 spectrophotometer (NanoDrop Technologies, Wilmington, DE, USA) and 4 ng of total RNA was reverse-transcribed using QuantiTect Reverse Transcription Kit (number 205314, Qiagen, Valencia, CA, USA). The reaction was made in a master mix including 8.0 *μ*L of total RNA (genomic DNA free), 1.0 *μ*L of Quantiscript Reverse Transcriptase, 4.0 *μ*L of Quantiscript RT Buffer, and 1 *μ*L of RT Primer Mix. The reaction was terminated by heating the samples at 95°C for 3 min.

For TH mRNA quantification, all samples were analyzed in triplicate in 10 *μ*L reaction, and a standard real-time PCR reaction mix was prepared containing the following components: 5.0 *μ*L of QuantiTect SYBR Green PCR Kit (number 204143, Qiagen, Valencia, CA, USA), 2.8 *μ*L of nanopure and sterile water, 0.1 *μ*L of each primer, and 2 *μ*L of cDNA. For specific gene amplification, a standard protocol of 45 cycles was used in a CFX96 Touch Real-Time PCR Detection System (Bio-Rad Laboratories, Inc., USA). After initial polymerase activation at 95°C for 10 min, primer-specific amplification and quantification cycles were run at 95°C for 15 sec and 61.7°C for 20 sec. The TH primer was designed from data published in GenBank, access number NM_012740: forward 5′-GGT-CTA-CTG-TCC-GCC-CGT-GAT-T-3′ and reverse 5′-GAG-CTT-GTC-CTT-GGC-GTC-ATT-G-3′. To normalize TH mRNA content, ribosomal 18s mRNA was measured in each protocol, using primers previously published [42] and commercially available, GenBank access number X01117 (for 18s, forward 5′-TCA AGA ACG AAA GTC GGA GG-3′ and reverse 5′-GGA CAT CTA AGG GCA TCA CA-3′). Amplification of 18s RNA was performed in a different tube to avoid interference with the amplification of the mRNAs. Reaction tubes lacking RT enzyme were used as PCR-negative controls. Specificity of generated amplicons was verified by performing melting curves at the end of each reaction. To verify the products from the RT-PCR reaction, they were separated on 2.0% agarose gels, stained with ethidium bromide, and compared to a 100 bp standard (data not shown).

### 2.5. Immunohistochemistry for Tyrosine Hydroxylase (TH)

Fifteen male rats were anesthetized with chloral hydrate (400 mg/Kg i.p.) and transcardially perfused with saline (0.9% p/v NaCl), followed by ice-cold fixative solution (4% p/v paraformaldehyde in phosphate buffered saline solution (PBS) 0.1 M with pH 7.4). Brains were removed from the skull and postfixed for 30 min. Brains were then dehydrated in 20% p/v sucrose solution for 48 h at 4°C. Afterwards, 30 *μ*m thick coronal slices were prepared on a cryostat (model KD-2950, Kedee, China). Interest slices were selected from SN-VTA located −5.4 mm from bregma according to the atlas of Paxinos and Watson [46]; these slices were put on 24-well plate and washed with PBS 0.01 M for 10 minutes following incubation with H_2_O_2_ (0.3% v/v in PBS) for 30 minutes.

Coronal slices were washed twice again with PBS 0.01 M for 10 minutes and then were incubated for 1 h in blocking solution (Triton X-100 0.4% v/v and NGS 3% v/v in PBS). The incubation of the first anti-TH rabbit antibody (catalog number 657012, Calbiochem, Merck Millipore, Merck KGaA, Darmstadt, Germany) it has been made with dilution 1 : 5000 in blocking solution over night with soft agitation at 4°C [47]. After incubation, slices were washed 4 times with PBS 0.01 M for 10 min each time. The second incubation it has been made for 2 h with second biotinilated anti-rabbit antibody (catalog number BA-1000, Vector Laboratory Inc., Burlingame, CA, USA) at ambient temperature, diluted 1 : 1000 in PBS 0.01 M solution with BSA 0.2% p/v. Afterwards, the slices were washed 4 times with the same protocol mentioned above. Then slices were incubated for 1 h with ABC kit (Kit Vectastain ABC, Vector Laboratory Inc., Burlingame, CA, USA) and then were washed twice. For the chromogenic stain, slices were incubated with DAB (diaminobenzidine) 0.05% p/v with H_2_O_2_ 0.025% v/v in PBS (DAB: catalog number D-5905, Sigma-Aldrich). At the end of the reaction (5–10 min), the slices were washed 2 times and finally were put on slide and fixed with Eukitt (catalog: 03989, Sigma-Aldrich, Inc., St. Louis, Missouri, USA).

The slices were photographed bilaterally with a microscope with Motic camera (BA-210, Motic, British Columbia, Canada) at 4x objective for area determination of SN and VTA; the following photographs were taken at 10x for TH-positive cells counting with ImageJ software (http://rsbweb.nih.gov/ij/). For each rat, SN and VTA were selected in 4 slices, then manual counts were performed in blind by three independent investigators using as reference the medial terminal nucleus of accessory optic tract (MT).

### 2.6. Dopamine Content in the SN-VTA

Tissue homogenization was performed according to Chi et al. [48] and our previous work [40]. Briefly, the tissue was collected in 400 *μ*L of 0.2 M perchloric acid and then homogenized in a glass-glass homogenizer in ice. The homogenate was centrifuged at 12,000 ×g for 15 minutes at 4°C (model Z233MK-2, Hermle LaborTechnik GmbH, Wehingen, Germany) and the resultant supernatant was filtered (0.2 *μ*m HPLC Syringe Filters disposable filter PTFE, model EW-32816-26, Cole-Parmer Instrument Company, USA). The filtered supernatant was injected into a HPLC coupled to electrochemical detection for determination of DA content. The pellet was resuspended in 1 N NaOH for protein quantification by the Bio-Rad Protein Assay (Bio-Rad Laboratories, Inc., Richmond, CA, USA) using bovine serum albumin as standard. The DA content was expressed as picograms per milligram of total protein.

### 2.7. *In Vivo* Brain Microdialysis

At PND 60, the animals were deeply anesthetized with choral hydrate (400 mg/Kg, i.p.) and placed in a stereotaxic apparatus (model 68002, RWD Life Science Co. Ltd., Shenzhen, China). Body temperature of the animals was maintained at 37°C with an electrical blanket controlled by a thermostat. A quarter of the initial dose of choral hydrate was given every hour to maintain the animal anesthetized during the course of the experiments. Concentric brain microdialysis probes (2 mm membrane length, model CMA 11, 6,000 Daltons cut-off, Solna, Sweden) were implanted in NAcc using the coordinates according to the atlas of Paxinos and Watson [41] (NAcc: 1.56 mm posterior, 1.50 mm lateral, and 7.8 mm ventral to bregma). Microdialysis probes were perfused with Krebs-Ringer's phosphate buffer (KRP in mM: NaCl 120; KCl 2.4; Na_2_HPO_4_ 0.9; NaH_2_PO_4_ 1.4; pH = 7.4) at a rate of 1 *μ*L/min using an infusion pump (model RWD 210, RWD Life Science Co. Ltd., Shenzhen, China). After a stabilization period of 90 min, two perfusion samples were collected every 20 min in 3 *μ*L of 0.2 M perchloric acid. At 40 min, KRP was changed for 70 mM KRP-potassium (K^+^) during 20 min. After those 20 min (between 60 and 100 min of perfusion protocol), KRP solution was again perfused through the microdialysis probe. All the perfusion samples were maintained on ice during the experiment and stored at −80°C until analysis. At the end of each experiment, animals were euthanized by decapitation and brains were quickly removed and stored in formalin. Brain sections of 50 *μ*m were stained with cresyl violet to verify microscopically probe location. One example of probe placement is shown in Figure 2(a).

### 2.8. DA and DOPAC Quantifications

Ten microliters of each cleaned supernatant or dialysate samples were injected to the HPLC system with the following setting: an isocratic pump (model PU-2080 Plus, Jasco Co. Ltd., Tokyo, Japan), a UniJet microbore column (MF-8912, BAS, West Lafayette, IN, USA), and an electrochemical detector (set at 650 mV, 0.5 nA; model LC-4C, BAS, West Lafayette, IN, USA). The mobile phase, containing 0.05 M NaH_2_PO_4_, 1.0 mM 1-octanesulfonic acid, 0.27 mM EDTA, and 4.0% (v/v) CH_3_CN (pH adjusted to 2.5), was pumped at a flow rate of 80 *μ*L/min. DA levels were assessed by comparing the respective peak area and elution time of the sample with a reference standard and the quantification was performed using a calibration curve for each neurotransmitter (Program ChromPass, Jasco Co. Ltd., Tokyo, Japan).

### 2.9. Statistical Analysis

Data were expressed as mean ± SEM. One-way ANOVA followed by Newman-Keuls post hoc test was used to determine eventual significant differences between groups. The statistical analyses were carried out with GraphPad Prism v5.0 (GraphPad Software, San Diego, CA).

## 3. Results 

The aim of our work was to determine if neonatal sex hormone exposure causes long-term changes in dopaminergic neural circuits associated with locomotion (nigrostriatal pathway) and motivation (reward system). This exposure to sex hormones during early developmental stages could be vulnerability factors predisposing to developing neuropsychiatric disorders in adulthood. To accomplish this aim, we used high performance liquid chromatography (HPLC) coupled to electrochemical detection to determine neurotransmitter levels in biological samples obtained from dissected brain (for tissue content) or microdialysates (for extracellular levels). We also used real-time PCR and immunohistochemistry to determine changes in gene and protein expression of TH.

### 3.1. Long-Lasting Effects of Neonatal Sex Hormones Administration on TH mRNA Expression in SN-VTA

**Figure 1:**
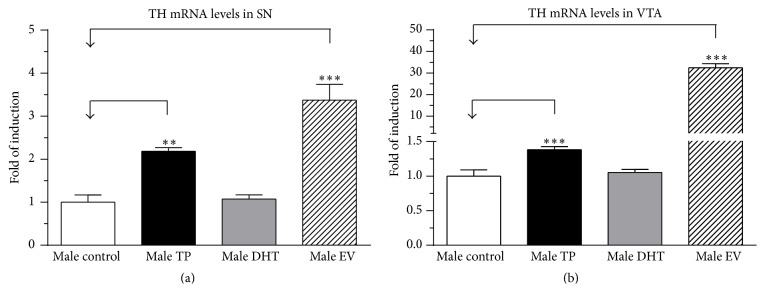
Effect of neonatal exposure to testosterone propionate (TP), dihydrotestosterone (DHT), or estradiol valerate (EV) on TH mRNA levels in substantia nigra (SN) (a) and ventral tegmental area (VTA) (b) of adult male rats. All data have been normalized for levels of 18s expression within the same sample. Results are expressed as fold of induction regard control group and represent the mean ± SEM (SN: *n* = 4, 4, 5, and 4 per male control, TP, DHT, and EV, resp.; VTA: *n* = 4, 3, 6, and 4 per male control, TP, DHT, and EV, resp.) (^*∗∗∗*^ *P* < 0.0001, ^*∗∗*^ *P* < 0.01; one-way ANOVA followed by the Newman-Keuls multiple comparison test).

In our model, neonatal exposures to EV and TP in male pups produced a significant increase in TH mRNA expression at PND 60 in SN (Figure 1(a) [*F*_(3,13)_ = 29.31, *P* < 0.0001]) and VTA (Figure 1(b) [*F*_(3,13)_ = 483.3, *P* < 0.0001]).

### 3.2. Long-Lasting Effects of Neonatal Sex Hormones Administration on TH Protein Expression in SN-VTA

**Figure 2:**
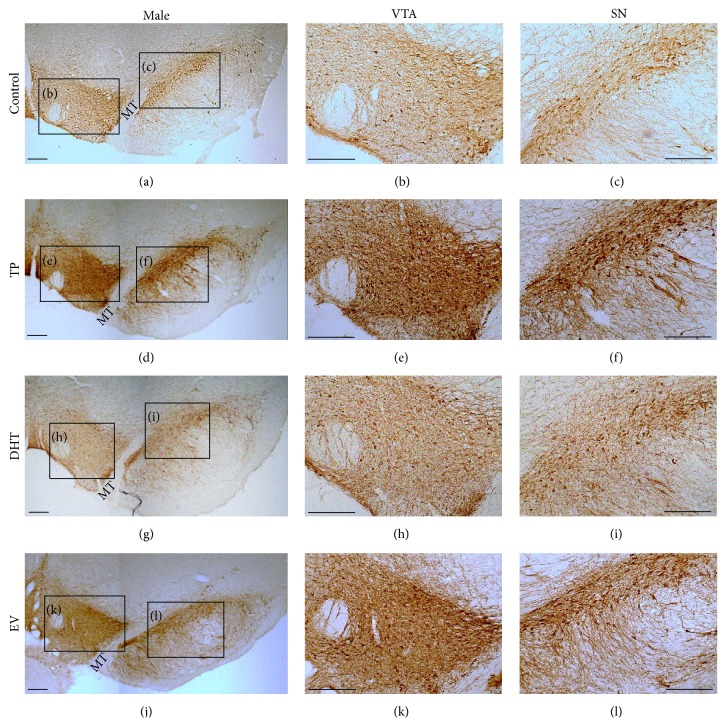
Effect of neonatal exposure to sesame oil (control: a, b, c), testosterone propionate (TP: d, e, f), dihydrotestosterone (DHT: g, h, i), or estradiol valerate (EV: j, k, l) on tyrosine hydroxylase-immunoreactive neurons in ventral tegmental area (VTA: b, e, h, j) and substantia nigra (SN: c, f, i, l) of adult male rats (control *n* = 3, TP = 4, DHT = 2 and EV = 5). Scale bars = 400 *μ*m. MT: medial terminal nucleus of accessory optic tract.

**Figure 3:**
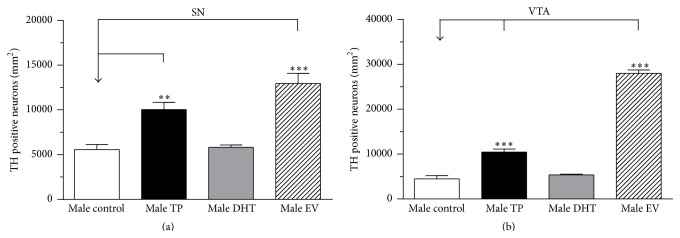
Quantitative analysis of tyrosine hydroxylase- (TH-) positive neurons in SN (a) and VTA (b) of adult male rats (PND 60) Data are expressed as the mean ± SEM (control *n* = 3, TP = 4, DHT = 3, and EV = 5) (^*∗∗∗*^ *P* < 0.0001, ^*∗∗*^ *P* < 0.01, and ^*∗*^ *P* < 0.05; one-way ANOVA followed by the Newman-Keuls multiple comparison test).

Figure 2 shows low and high magnification photomicrographs of TH-immunoreactive neurons in SN and VTA of control, TP, DHT, and EV male rats. Neonatal exposures to EV and TP increase TH protein expression at PND 60 in SN (Figure 3(a) [*F*_(3,11)_ = 15.22, *P* = 0.0003]) and VTA (Figure 3(b) [*F*_(3,11)_ = 280.4, *P* < 0.0001]).

### 3.3. Long-Lasting Effects of Neonatal Sex Hormones Administration on DA and DOPAC Content in SN-VTA and Striatum

**Figure 4:**
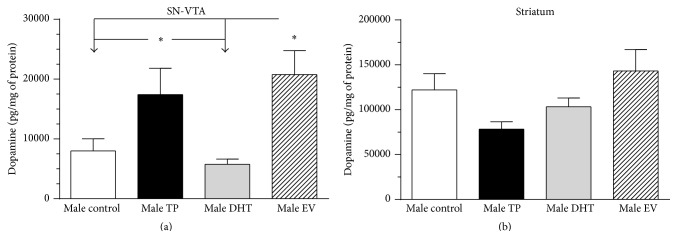
Dopamine content in the substantia nigra-ventral tegmental area (SN-VTA) (a) and striatum (b) of adult male with neonatal exposure to testosterone propionate (TP), dihydrotestosterone (DHT), or estradiol valerate (EV). Results are expressed as pg/mg of protein and represent the mean ± SEM (^*∗*^ *P* < 0.05; one-way ANOVA followed by the Newman-Keuls multiple comparison test; *n* = 6 per male in each condition).

Figure 4 shows the effects of neonatal administration of TP, DHT, or EV on DA content in SN-VTA (panel (a)) and striatum (panel (b)) of adult male rats. Neonatal exposure to TP or EV produced a significant increase on DA content in SN-VTA of adult male rats [*F*_(3,20)_ = 5.206, *P* = 0.0081]. However, the neonatal administration of DHT (a nonaromatizable androgen) did not affect the DA content (Figure 4). On the other hand, striatal DA content was unaffected by neonatal sex hormones administration compared to control rats (Figure 4(b)) [*F*_(3,20)_ = 2.884, *P* = 0.0613].

When we analyzed the ratio between the main metabolite of DA (DOPAC) and DA in SN-VTA, we observed a significant reduction in this value in EV versus control rats (Table 1, ^*∗*^ *P* < 0.05). On the contrary, the ratio (DOPAC/DA) in striatum increases significantly in DHT versus control rats (Table 2, ^*∗*^ *P* < 0.0001).

### 3.4. Long-Lasting Effects of Neonatal Sex Hormones Administration on DA Release in NAcc

**Figure 5:**
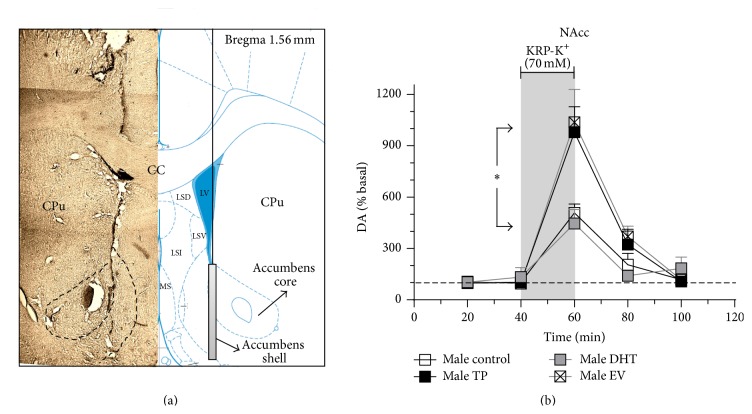
Dopamine release in nucleus accumbens (NAcc) of adult male with neonatal exposure to testosterone propionate (TP), dihydrotestosterone (DHT), or estradiol valerate (EV). Panel (a) (left) shows a typical placement of microdialysis probe in NAcc. Panel (a) (right) shows a scheme of NAcc extracted from the rat brain atlas [41] with an example of the theoretical probe position. Panel (b) shows extracellular dopamine levels in the NAcc and the grey bar indicates the time during which Krebs-Ringer's phosphate (KRP) buffer-K^+^ [70 mM] was perfused. Asterisk indicates a significant difference when comparing the effect of KRP-K^+^between the respective experimental groups (^*∗*^ *P* < 0.05; one-way ANOVA followed by the Newman-Keuls multiple comparison test). Results are expressed as percentage of the respective basal levels (mean) ± SEM (*n* = 6 for male control and *n* = 5 for each treatment group). MS, medial septum; LSI, lateral septum intermediate; LSV, lateral septum ventral; LSD, lateral septum dorsal; cc, corpus callosum; LV, lateral ventricle; CPu, caudate putamen.

Figure 5(b) shows the effects of neonatal exposure to TP, DHT, or EV on NAcc DA release induced by depolarizing stimulus in adult male rats. For each experimental group, the perfusion of KRP-K^+^ produced an increase in NAcc DA release with respect to its own baseline levels. However, when comparing the magnitudes of the NAcc DA release induced by KRP-K^+^ at 60 min, we observed a greater effect in DA releasability in TP and EV rats versus DHT and control rats [*F*_(3,17)_ = 6.031, *P* = 0.0054].

## 4. Discussion

### 4.1. Long-Lasting Effects of Neonatal Sex Hormones Administration on mRNA and Protein Expression of TH and DA Content in SN-VTA

Our work demonstrates that DA content in SN-VTA in adult male rats is affected by early exposure to E_2_—either directly by EV exposure or indirectly through the partial aromatization of T to E_2_ in TP male—which produces an increase in mRNA and protein expression of TH (Figures 1–3), which results in an increase in DA content in SN-VTA. The fact that we did not find similar effects with neonatal exposure to DHT validates our “aromatization hypothesis.” Thus, present results show that DHT, a nonaromatizable androgen, did not cause changes in the CNS parameters measured but that there were long-lasting effects observed in peripheral tissues. Although the doses of TP or DHT (1 mg/Kg s.c.) used in our study could be considered high, to the early life stage, similar doses have been previously reported in previous studies by our [39] and other research groups [49, 50], aimed at triggering evident neuroendocrine changes in the brain.

Our proposed “aromatization hypothesis” for neonatal exposure to TP at PND 1 in males rats is based on the high levels of cytochrome P450 aromatase expression and activity in rat midbrain [51], which would promote the conversion of T to E_2_. Although it has been shown that E_2_, through binding to estrogen receptors (ERs), is able to increase TH expression in midbrain dopaminergic neurons of adult female rodents [13, 27–29], it is unlikely that testosterone and estradiol administered at PND 1 remain in serum until PND 60 and cause a direct effect on dopaminergic neurons in adulthood. Therefore, TP and EV increased estradiol levels in the brain within an early window of development, which indeed could alter the patterns of neurogenesis and apoptosis of neural dopaminergic progenitors through modulating neurotrophic factors (for review see [28]). This probably is the cause for the increased number of TH-positive neurons demonstrated in our study. In addition to this, we also observed an increase in TH expression in adulthood. This is consistent with the fact that ER*α* knockout mice have decreased TH protein levels in midbrain dopaminergic neurons [52] compared to wild type mice. The regulation of TH by estrogens occurs mainly by the genomic pathway and involves the binding of the ligand-receptor complex to estrogen response elements in the TH gene [53]. We think that the long-term effect of this early increase in serum estradiol on TH expression could involve long-term epigenetic regulation of gene expression, as observed in multiple genes in different models of estrogenic exposure [54]. However we cannot exclude the possibility that the neonatal administration of TP or EV may cause permanent changes in the cytochrome P450 aromatase expression in the brain or at testicular level such as previously demonstrated by Persky et al. [55].

The increased mRNA and protein expression of TH observed in our work could be responsible for the increased DA content observed in SN-VTA in adult male rats exposed early to EV or TP (Figure 4). In this regard, the magnitude of the increased TH expression in TP rats was lower than in EV rats, possibly due to the partial aromatization of exogenous T to E_2_. In this sense, we previously demonstrated that neonatal exposure to EV increases DA content in the ventromedial hypothalamus [39] and SN-VTA in adult female rats [40].

### 4.2. Long-Lasting Effects of Neonatal Sex Hormones Administration on DA Release in NAcc

Dopamine release induced by a depolarizing stimulus in NAcc of adult male rats exposed to EV and TP during first hours of life was greater than in DHT and control rats. This effect is directly related to the high mRNA and protein expression of TH (Figures 1, 2, and 3) and DA content (Figure 4(a)) in SN-VTA of EV and TP rats. As discussed previously, greater NAcc DA release induced by KRP-K^+^ perfusion is produced by neonatal exposure to high levels of E_2_ that programs midbrain dopaminergic neurons in adult male rats.

Another possibility to explain the effect of early sex hormone administration on increased DA release has been suggested from* in vitro* experiments using superfusion chambers with slices of hypothalamus. Becker and Ramirez showed an increase of amphetamine-induced DA release in hypothalamus slices in castrated adult male rats compared to castrated adult male rats supplemented with TP [30]. This is an interesting finding since it has been shown that castrated adult male rats have higher TH expression levels in SN-VTA than intact male rats [33] or castrated male rats with TP replacement [30]. In this sense, we observed a reduction in T serum levels in adult male rats treated neonatally with TP, EV, or DHT, which could be associated with a reduction in the size of the testes in the adulthood (see Supplementary Figure  1B in Supplementary Material available online at http://dx.doi.org/10.1155/2016/4569785). Possibly the reduction of T serum levels observed in adult males treated neonatally with DHT is due to the high doses of DHT used by us, since, in the work of Persky et al., they used lower doses of DHT and did not observe changes in T serum T levels in the adulthood [55]. This study suggests that early exposure to EV or TP produces an increase in NAcc DA release induced by depolarizing stimulus through an estrogenic effect produced by neonatal exposure to E_2_ or by and increased endogenous production of E_2_ in adulthood as demonstrated by Persky et al. using the neonatal administration of TP [55].

At the behavioral level, we previously reported that, in EV female rats, a single dose of amphetamine (1 mg/Kg i.p.) did not produce a significant increase in locomotor activity when compared with control female rats [40]. This is interesting, as in the current study we observed a reduction in the ratio of DOPAC to DA in male rats that could reflect a reduction in DA uptake, suggesting a reduction in DAT levels. In this sense, DAT is the main pharmacologic target of amphetamine and a reduction in DAT expression could be related with a reduction in pharmacological effect of amphetamine. In the literature, this hypothesis is supported in a reduction of DAT expression in the NAcc of ovariectomized rats [56] and a reduction of DAT expression in striatum of rats exposed to BPA during prenatal and postnatal stages [57].

Neonatal exposure to sex hormones, particularly during critical periods of development, may induce long-lasting changes in the CNS. In this sense, E_2_ administered in the neonatal period shows an important effect on dopaminergic circuitry; as demonstrated in the current work, a single dose of EV or TP increases DA content and TH expression (limiting enzyme of DA synthesis) in mesolimbic areas. These changes also involve an increase in DA release in NAcc. These effects are produced by a positive modulation on TH expression produced by estrogens, suggesting that there may be a role of aromatization of E_2_ to T, as a nonaromatizable androgen such as DHT did not produce the same effect. We strongly think that our “aromatization hypothesis” could be ratified in future studies by using the coadministration of a selective cytochrome P450 aromatase inhibitor such as letrozole or anastrozole along with TP.

Finally, neonatal administration of estradiol, or testosterone through its aromatization to E_2_, produced long-lasting effects in midbrain dopaminergic neurons that are associated with persistent alteration of sex hormones. The current results are of importance, as exposure to hormone-like compounds in the environment during critical developmental periods could have long-term effects on brain function. In this sense, different environmental pollutants acting as endocrine-disrupting chemicals could permanently modify neurotransmitter levels in the brain and produce long-lasting changes. Further research in this arena is warranted, provided the involvement of dopaminergic circuits in Parkinson's disease, psychiatric disorders, and addiction.

## Supplementary Material

Supplementary material shows the effects of neonatal exposure to sex hormones on testes size and testosterone serum levels.

## Figures and Tables

**Table 1:** Substantia nigra-ventral tegmental area (SN-VTA) DA and DOPAC levels from control, TP, DHT, and EV adult male rats.

Group	DA (pg/mg protein)	SEM	DOPAC (pg/mg protein)	SEM	DOPAC/DA	SEM	*P* value	*n*
C	7985.3	2029.6	2450.9	603.6	0.3532	0.0491	—	6
TP	17407.5	4388.6	3175.9	538.5	0.2913	0.0654	0.457	6
DHT	5765.8	858.8	1889.2	277.3	0.3121	0.0329	0.574	6
EV	20744.0	4007.3	4109.1	635.3	0.2117	0.0208	0.030^*∗*^	6

DA: dopamine; DOPAC: 3,4-dihydroxiphenylacetic acid. ^*∗*^ *P* value < 0.05 when comparing the ratio (DOPAC/DA) to the control group.

**Table 2:** Striatal DA and DOPAC levels from control, TP, DHT, and EV adult male rats.

Group	DA (pg/mg protein)	SEM	DOPAC (pg/mg protein)	SEM	DOPAC/DA	SEM	*P* value	*n*
C	122001	18034	12970	2854	0.1004	0.014	—	7
TP	78322	8136	10714	1706	0.1359	0.013	0.0875	6
DHT	103276	9689	27071	2737	0.2707	0.023	0.0001^*∗*^	6
EV	143159	23860	15583	3034	0.1156	0.024	0.5790	6

DA: dopamine; DOPAC: 3,4-dihydroxiphenylacetic acid. ^*∗*^ *P* value < 0.05 when comparing the ratio (DOPAC/DA) to the control group.

## References

[1] Lucas A. (1991). Programming by early nutrition in man. *Ciba Foundation Symposium*.

[2] Phoenix C. H., Goy R. W., Gerall A. A., Young W. C. (1959). Organizing action of prenatally administered testosterone propionate on the tissues mediating mating behavior in the female guinea pig. *Endocrinology*.

[3] McIntyre M. H. (2006). The use of digit ratios as markers for perinatal androgen action. *Reproductive Biology and Endocrinology*.

[4] McIntyre M. H., Cohn B. A., Ellison P. T. (2006). Sex dimorphism in digital formulae of children. *American Journal of Physical Anthropology*.

[5] Panzica G. C., Bo E., Martini M. A. (2011). Neuropeptides and enzymes are targets for the action of endocrine disrupting chemicals in the vertebrate brain. *Journal of Toxicology and Environmental Health—Part B: Critical Reviews*.

[6] Schantz S. L., Widholm J. J. (2001). Cognitive effects of endocrine-disrupting chemicals in animals. *Environmental Health Perspectives*.

[7] Viberg H., Fredriksson A., Eriksson P. (2007). Changes in spontaneous behaviour and altered response to nicotine in the adult rat, after neonatal exposure to the brominated flame retardant, decabrominated diphenyl ether (PBDE 209). *NeuroToxicology*.

[8] Ishido M., Yonemoto J., Morita M. (2007). Mesencephalic neurodegeneration in the orally administered bisphenol A-caused hyperactive rats. *Toxicology Letters*.

[9] Randall V. A. (1994). 9 Role of 5*α*-reductase in health and disease. *Bailliere's Clinical Endocrinology and Metabolism*.

[10] Lephart E. D. (1997). Molecular aspects of brain aromatase cytochrome P450. *Journal of Steroid Biochemistry and Molecular Biology*.

[11] Celotti F., Melcangi R. C., Martini L. (1992). The 5 alpha-reductase in the brain: molecular aspects and relation to brain function. *Frontiers in Neuroendocrinology*.

[12] Celotti F., Negri-Cesi P., Poletti A. (1997). Steroid metabolism in the mammalian brain: 5alpha-reduction and aromatization. *Brain Research Bulletin*.

[13] Beyer C. (1999). Estrogen and the developing mammalian brain. *Anatomy and Embryology*.

[14] Morikawa H., Paladini C. A. (2011). Dynamic regulation of midbrain dopamine neuron activity: intrinsic, synaptic, and plasticity mechanisms. *Neuroscience*.

[15] Ungerstedt U. (1968). 6-hydroxy-dopamine induced degeneration of central monoamine neurons. *European Journal of Pharmacology*.

[16] Glick S. D., Greenstein S. (1973). Possible modulating influence of frontal cortex on nigro-striatal function. *British Journal of Pharmacology*.

[17] German D. C., Manaye K. F. (1993). Midbrain dopaminergic neurons (nuclei A8, A9, and A10): three-dimensional reconstruction in the rat. *Journal of Comparative Neurology*.

[18] Chinta S. J., Andersen J. K. (2005). Dopaminergic neurons. *International Journal of Biochemistry and Cell Biology*.

[19] Gysling K., Wang R. Y. (1983). Morphine-induced activation of A10 dopamine neurons in the rat. *Brain Research*.

[20] Koob G. F., Volkow N. D. (2010). Neurocircuitry of addiction. *Neuropsychopharmacology*.

[21] Hearing M. C., Zink A. N., Wickman K. (2012). Cocaine-induced adaptations in metabotropic inhibitory signaling in the mesocorticolimbic system. *Reviews in the Neurosciences*.

[22] Wise R. A. (2002). Brain reward circuitry: insights from unsensed incentives. *Neuron*.

[23] Pfaus J. G., Damsma G., Nomikos G. G. (1990). Sexual behavior enhances central dopamine transmission in the male rat. *Brain Research*.

[24] Bassareo V., Di Chiara G. (1997). Differential influence of associative and nonassociative learning mechanisms on the responsiveness of prefrontal and accumbal dopamine transmission to food stimuli in rats fed ad libitum. *Journal of Neuroscience*.

[25] Di Chiara G., Imperato A. (1988). Drugs abused by humans preferentially increase synaptic dopamine concentrations in the mesolimbic system of freely moving rats. *Proceedings of the National Academy of Sciences of the United States of America*.

[26] Creutz L. M., Kritzer M. F. (2004). Mesostriatal and mesolimbic projections of midbrain neurons immunoreactive for estrogen receptor beta or androgen receptors in rats. *The Journal of Comparative Neurology*.

[27] Küppers E., Ivanova T., Karolczak M., Lazarov N., Föhr K., Beyer C. (2001). Classical and nonclassical estrogen action in the developing midbrain. *Hormones and Behavior*.

[28] Kipp M., Karakaya S., Pawlak J., Araujo-Wright G., Arnold S., Beyer C. (2006). Estrogen and the development and protection of nigrostriatal dopaminergic neurons: concerted action of a multitude of signals, protective molecules, and growth factors. *Frontiers in Neuroendocrinology*.

[29] Johnson M. L., Ho C. C., Day A. E., Walker Q. D., Francis R., Kuhn C. M. (2010). Oestrogen receptors enhance dopamine neurone survival in rat midbrain. *Journal of Neuroendocrinology*.

[30] Becker J. B., Ramirez V. D. (1981). Sex differences in the amphetamine stimulated release of catecholamines from rat striatal tissue in vitro. *Brain Research*.

[31] Radcliffe P. M., Sterling C. R., Tank A. W. (2009). Induction of tyrosine hydroxylase mRNA by nicotine in rat midbrain is inhibited by mifepristone. *Journal of Neurochemistry*.

[32] Raab H., Pilgrim C., Reisert I. (1995). Effects of sex and estrogen on tyrosine hydroxylase mRNA in cultured embryonic rat mesencephalon. *Molecular Brain Research*.

[33] Johnson M. L., Day A. E., Ho C. C., Walker Q. D., Francis R., Kuhn C. M. (2010). Androgen decreases dopamine neurone survival in rat midbrain. *Journal of Neuroendocrinology*.

[34] Dluzen D. E. (2000). Neuroprotective effects of estrogen upon the nigrostriatal dopaminergic system. *Journal of Neurocytology*.

[35] Lenz B., Müller C. P., Stoessel C. (2012). Sex hormone activity in alcohol addiction: integrating organizational and activational effects. *Progress in Neurobiology*.

[36] Baron-Cohen S., Auyeung B., Nørgaard-Pedersen B. (2015). Elevated fetal steroidogenic activity in autism. *Molecular Psychiatry*.

[37] King J. A., Barkley R. A., Delville Y., Ferris C. F. (2000). Early androgen treatment decreases cognitive function and catecholamine innervation in an animal model of ADHD. *Behavioural Brain Research*.

[38] Sundblad C., Eriksson E. (1997). Reduced extracellular levels of serotonin in the amygdala of androgenized female rats. *European Neuropsychopharmacology*.

[39] Sotomayor-Zárate R., Tiszavari M., Cruz G., Lara H. E. (2011). Neonatal exposure to single doses of estradiol or testosterone programs ovarian follicular development-modified hypothalamic neurotransmitters and causes polycystic ovary during adulthood in the rat. *Fertility and Sterility*.

[40] Cruz G., Riquelme R., Espinosa P. (2014). Neonatal exposure to estradiol valerate increases dopamine content in nigrostriatal pathway during adulthood in the rat. *Hormone and Metabolic Research*.

[41] Paxinos G., Watson C. (2005). *The Rat Brain in Stereotaxic Coordinates*.

[42] Sotomayor-Zárate R., Dorfman M., Paredes A., Lara H. E. (2008). Neonatal exposure to estradiol valerate programs ovarian sympathetic innervation and follicular development in the adult rat. *Biology of Reproduction*.

[43] Barraclough C. A. (1961). Production of anovulatory, sterile rats by single injections of testosterone propionate. *Endocrinology*.

[44] Torrens Y., Beaujouan J. C., Besson M. J., Michelot R., Glowinski J. (1981). Inhibitory effects of GABA, L-glutamic acid and nicotine on the potassium-evoked release of substance P in substantia nigra slices of the rat. *European Journal of Pharmacology*.

[45] Abarca J., Bustos G. (1999). Differential regulation of glutamate, aspartate and *γ*-amino-butyrate release by *N*-methyl-D-aspartate receptors in rat striatum after partial and extensive lesions to the nigro-striatal dopamine pathway. *Neurochemistry International*.

[46] Paxinos G., Watson C. (2009). *The Rat Brain in Stereotaxic Coordinates*.

[47] Aliaga E., Cárcamo C., Abarca J., Tapia-Arancibia L., Bustos G. (2000). Transient increase of brain derived neurotrophic factor mRNA expression in substantia nigra reticulata after partial lesion of the nigrostriatal dopaminergic pathway. *Molecular Brain Research*.

[48] Chi J. D., Odontiadis J., Franklin M. (1999). Simultaneous determination of catecholamines in rat brain tissue by high-performance liquid chromatography. *Journal of Chromatography B: Biomedical Sciences and Applications*.

[49] Jacobson C. D., Csernus V. J., Shryne J. E., Gorski R. A. (1981). The influence of gonadectomy, androgen exposure, or a gonadal graft in the neonatal rat on the volume of the sexually dimorphic nucleus of the preoptic area. *The Journal of Neuroscience*.

[50] Sakuma Y. (1984). Influences of neonatal gonadectomy or androgen exposure on the sexual differentiation of the rat ventromedial hypothalamus. *The Journal of Physiology*.

[51] Raab H., Beyer C., Wozniak A., Hutchison J. B., Pilgrim C., Reisert I. (1995). Ontogeny of aromatase messenger ribonucleic acid and aromatase activity in the rat midbrain. *Molecular Brain Research*.

[52] Küppers E., Krust A., Chambon P., Beyer C. (2008). Functional alterations of the nigrostriatal dopamine system in estrogen receptor-*α* knockout (ERKO) mice. *Psychoneuroendocrinology*.

[53] Maharjan S., Serova L., Sabban E. L. (2005). Transcriptional regulation of tyrosine hydroxylase by estrogen: opposite effects with estrogen receptors *α* and *β* and interactions with cyclic AMP. *Journal of Neurochemistry*.

[54] Cruz G., Foster W., Paredes A., Yi K. D., Uzumcu M. (2014). Long-term effects of early-life exposure to environmental oestrogens on ovarian function: role of epigenetics. *Journal of Neuroendocrinology*.

[55] Persky R. W., Liu F., Xu Y. (2013). Neonatal testosterone exposure protects adult male rats from stroke. *Neuroendocrinology*.

[56] Chavez C., Hollaus M., Scarr E., Pavey G., Gogos A., van den Buuse M. (2010). The effect of estrogen on dopamine and serotonin receptor and transporter levels in the brain: an autoradiography study. *Brain Research*.

[57] Tian Y.-H., Baek J.-H., Lee S.-Y., Jang C.-G. (2010). Prenatal and postnatal exposure to bisphenol A induces anxiolytic behaviors and cognitive deficits in mice. *Synapse*.

