# Supplementary figures and images for: Programming of Dopaminergic Neurons by Neonatal Sex Hormone Exposure: Effects on Dopamine Content and Tyrosine Hydroxylase Expression in Adult Male Rats
Source: Neural Plast. 2016 Jan 10;2016:4569785. doi: 10.1155/2016/4569785 (PMC4745917; doi:10.1155/2016/4569785)

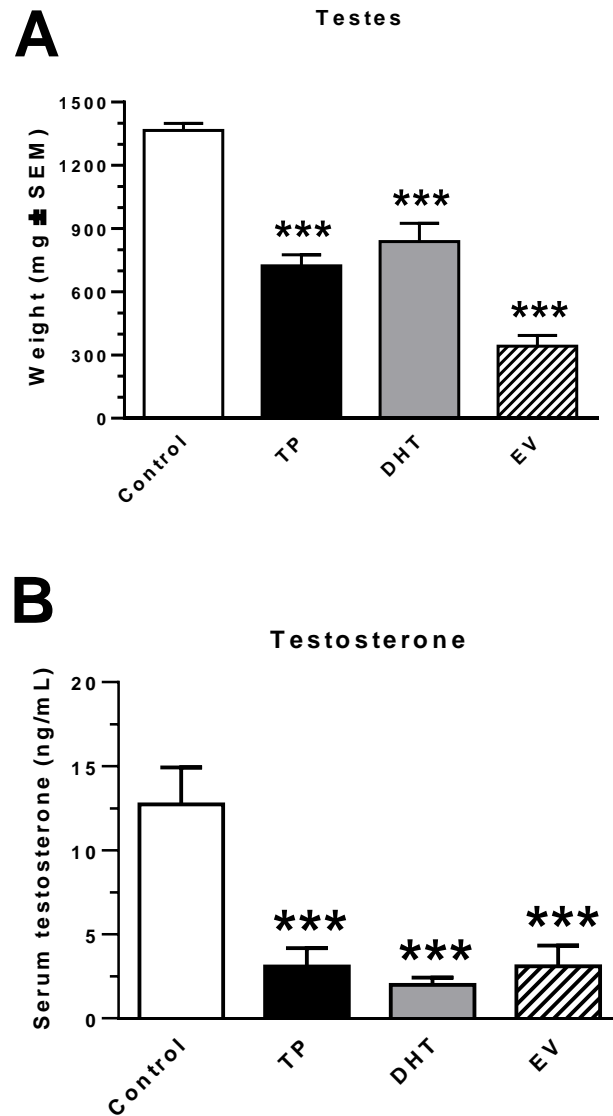

Supplement: Supplementary file 1 — Supplementary material shows the effects of neonatal exposure to sex hormones on testes size and testosterone serum levels. [file 4569785.F1.pdf]
